# 

**Published:** 1881-01

**Authors:** 


Vol. II.] TWO DOLLARS A YEAR. SINGLE COPIES 50 CENTS. [No 1

### THE JOURNAL
(FORMERLY ARCHIVES)
OF
### COMPARATIVE MEDICINE AND SURGERY.

### A Quarterly Journal
OF THE
### ANATOMY, PATHOLOGY, AND THERAPEUTICS OF THE LOWER ANIMALS.

NEW YORK:
W. L. HYDE & CO., PUBLISHERS, NO. 22 UNION SQUARE.
1881.

